# Impact of Complete Surgical Resection of Metastatic Lesions in Patients with Advanced Renal Cell Carcinoma in the Era of Tyrosine Kinase Inhibitors and Immune Checkpoint Inhibitors

**DOI:** 10.3390/cancers16040841

**Published:** 2024-02-19

**Authors:** Takuto Shimizu, Makito Miyake, Nobutaka Nishimura, Takanori Yoshida, Yoshitaka Itami, Akira Tachibana, Chihiro Omori, Yuki Oda, Mikiko Kohashi, Mitsuru Tomizawa, Kenta Onishi, Shunta Hori, Yosuke Morizawa, Daisuke Dotoh, Yasushi Nakai, Kazumasa Torimoto, Nobumichi Tanaka, Kiyohide Fujimoto

**Affiliations:** 1 Department of Urology, Nara Medical University, 840 Shijo-cho, Kashihara, Nara 634-8522, Japan; takutea19@gmail.com (T.S.); tigers.yosuke@gmail.com (Y.M.);; 2 Department of Urology, Nara Prefecture Seiwa Medical Center, Ikoma, Nara 636-0802, Japan; 3 Department of Urology, Tane General Hospital, Osaka, Osaka 550-0025, Japan; 4 Department of Urology, Hoshigaoka Medical Center, Hirakata, Osaka 573-8511, Japan; 5 Department of Urology, Nara Prefecture General Medical Center, Nara, Nara 630-8581, Japan; 6 Department of Urology, Nara City Hospital, Nara, Nara 630-8305, Japan; 7 Department of Prostate Brachytherapy, Nara Medical University, 840 Shijo-cho, Kashihara, Nara 634-8522, Japan

**Keywords:** renal cell carcinoma, metastasectomy, immune checkpoint inhibitor, tyrosine kinase inhibitor

## Abstract

**Simple Summary:**

This study investigated the efficacy of complete metastasectomy (CM) in metastatic renal cell carcinoma (mRCC) during the tyrosine kinase inhibitor (TKI) and immune checkpoint inhibitor (ICI) era. Analyzing data from a multi-institutional database with 367 mRCC patients, the CM group exhibited significantly longer overall survival than the non-CM group in unadjusted cohorts (*p* < 0.001, hazard ratio 0.49, 95% confidence interval 0.35–0.69). However, this superiority was not sustained in adjusted cohorts. The median disease-free survival (DFS) after CM was 24 months, with no significant differences noted based on the time of relapse. This study supports CM’s potential in mRCC management during the TKI/ICI era, acknowledging limitations such as sample size and selection bias.

**Abstract:**

Complete metastasectomy (CM) in metastatic renal cell carcinoma (mRCC) has demonstrated efficacy in the cytokine era, but its effectiveness in the era of tyrosine kinase inhibitors (TKIs) and immune checkpoint inhibitors (ICIs) remains unclear. A multi-institutional database included clinicopathological data of 367 patients with mRCC. Patients were divided into two groups: the CM group and the non-CM group. These two groups were compared before and after propensity score matching (PSM). Cox proportional hazard models were used to detect factors associated with disease-free survival (DFS) and overall survival (OS) from mRCC diagnosis. The CM group showed a significant association with longer overall survival compared to the non-CM group in the PSM-unadjusted cohorts (*p* < 0.001, hazard ratio 0.49, 95% confidence interval 0.35–0.69), but no superiority was noted in the adjusted cohorts. The median DFS after CM was 24 months, with no significant differences based on relapse timing. Notably, the international metastatic RCC database consortium risk categories and metastatic burden were associated with DFS. This study supports the potential of CM in mRCC management during the TKI/ICI era, although limitations including sample size and selection bias need to be considered.

## 1. Introduction

Renal cell carcinomas (RCCs) are clinically heterogeneous, ranging from extensive and fast progressing to those with a slow course with few metastases [1]. Modern immune checkpoint inhibitor (ICI)-based combination therapies have a median progression-free survival of 12–24 months, are costly and cause a high rate of severe toxicity (grade 3 or above) in 46–82% of patients [2,3,4,5,6]. These trials made important advances and prolonged survival, but the benefits were mainly focused on diseases with a relatively rapid clinical course, such as intermediate and poor in the international metastatic RCC database consortium (IMDC)’s risk classification. In cases of favorable risk, the benefit is limited. For patients with a low-grade clinical presentation and a small number of metastatic sites (i.e., oligometastasis), reliable local control of metastases is considered an option that can delay or even eliminate the need for systemic therapy. Complete metastasectomy (CM) for metastatic lesion of RCC has been shown to have an oncological benefit to some extent for appropriately selected patients [7,8,9,10], and each guideline has a certain level of consensus, although the level of evidence is not high [11,12]. There have been no prospective randomized trials on metastatic resection. Systematic reviews of retrospective studies [8,9] have reported benefits in the overall survival (OS) and cancer-specific survival (CSS), and poor prognostic factors after metastatic resection include incomplete resection, primary tumor T3 or greater, a Fuhrman grade 3 or greater, extrapulmonary metastases and multiple metastases, although most of these are based on data before the advent of tyrosine kinase inhibitors (TKIs) or ICIs. There is little data to show whether CM is still an effective strategy after 2008, that is, after the approval of TKIs and ICIs [13,14].

Pembrolizumab was recently approved for adjuvant setting after radical surgery in RCC with a high risk of recurrence and M1 no evidence of disease (M1NED) after radical surgery of the primary lesion and CM of metastases based on the positive results of the KEYNOTE 564 clinical trial [15,16]. A sub-analysis of the M1NED in the KEYNOTE 564 trial demonstrated a remarkable benefit from adjuvant pembrolizumab. Conversely, the IMmotion010 clinical trial failed to show a benefit of adjuvant setting of atezolizumab in M1NED RCC [17]. A critical distinction between these two studies may lie in the patient eligibility. KEYNOTE 564 exclusively focused on patients with a metachronous interval of <1 year following primary surgery, while Immotion010 included individuals who underwent CM for metastases occurring ≥1 year after the primary diagnosis. According to the IMDC classification, a metachronous interval <1 year for recurrence after surgery with curative intent is a poor prognostic factor. It is unclear whether pembrolizumab after CM or ICI-based combination therapy is more effective in this high-risk subgroup.

Therefore, we conducted a multi-institutional retrospective study to investigate the clinical benefit of CM, including the extent to which systemic treatment can be avoided or extended, and whether there are differences in the oncological outcomes after CM in the recurrence <1 year and recurrence ≥1 year in the TKI/ICI era.

## 2. Materials and Methods

### 2.1. Patient Selection

This multi-institutional retrospective study was approved by the Institutional Review Board (IRB) of Nara Medical University (Nara, Japan; study protocol ID: NMU-2891) and complied with the 1964 Helsinki Declaration and its later amendments. The study was also approved by the ethics committee of each participating institute. As the data for the study were obtained through a retrospective review, informed consent was obtained from participants through posters and/or websites using the opt-out method.

### 2.2. Data Collection

The study included a total of 386 patients who were diagnosed with metastatic RCC (mRCC) between 2008 and 2022. The clinicopathological data and follow-up data were collected via a retrospective chart review. Clinicopathological characteristics included age, gender, time from first RCC diagnosis, IMDC risk classification, metastatic site and number of metastases at the time of first diagnosis of distant (i.e., index) metastasis. Also included were the presence or absence of nephrectomy, RCC subtype and Fuhrman Grade. Follow-up data, including clinical outcomes and survival, were calculated from the diagnosis of mRCC to the last documented follow-up or data lock (April 2022).

Imaging to assess metastases is not standardized. However, computed tomography (CT) and Magnetic Resonance Imaging (MRI) are commonly used. Brain CT or MRI is performed when clinically indicated. CM was defined in this study as complete surgical resection within 90 days of the diagnosis of metastasis without systemic drug therapy or other local therapy for the metastasis. CM procedure was performed by a specialist surgeon at each metastatic site (e.g., respiratory surgery for lung metastases, orthopedic surgery for bone metastases, neurosurgery for brain metastases). All patients who underwent CM surgery were also included as cases in which local renal lesions were also resected. In addition, since this patient cohort was before the approval of the postoperative adjuvant Pembrolizumab, not all patients received postoperative adjuvant therapy after CM.

### 2.3. Statistical Analysis

Statistical analyses were conducted using GraphPad Prism 5.0 (GraphPad Software, San Diego, CA, USA) for certain aspects, while propensity score matching (PSM) analysis was implemented via EZR, a graphical user interface for R, developed jointly by Jichi Medical University and Saitama Medical University’s Statistical Computing Centre. The utilization of PSM methodology was integral to minimize potential biases that are inherent in observational studies by balancing covariates between treatment groups. Clinicopathological characteristics were assessed through various statistical tests tailored to the nature of the data. Continuous variables were analyzed using the Mann–Whitney U test, whereas categorical variables were evaluated via the Chi-square test or Fisher’s direct probability test, as deemed appropriate. The baseline characteristics were matched by calculating the propensity score for each group using a multivariable logistic regression model based on covariates, such as age, time from diagnosis to treatment, presence or absence of primary tumor removal, and number of metastases; these were significantly different between the two groups before PSM. The baseline characteristics were matched by calculating the propensity score for each patient using a multivariable logistic regression model based on covariates. As a result, a nearly balanced distribution of baseline covariates between the two groups was ensured after PSM. Survival analysis was conducted to assess overall survival (OS) and disease-free survival (DFS) outcomes. The Kaplan–Meier method was employed to estimate survival curves, offering a comprehensive visualization of the temporal trends in survival probabilities over the study period. Furthermore, the log-rank test was utilized to compare survival distributions between distinct groups, facilitating the identification of statistically significant differences in survival outcomes. The threshold for statistical significance was established at *p* < 0.05, employing a two-tailed test to comprehensively evaluate the hypotheses under scrutiny.

## 3. Results

### 3.1. Cohort of This Study

**Figure 1:**
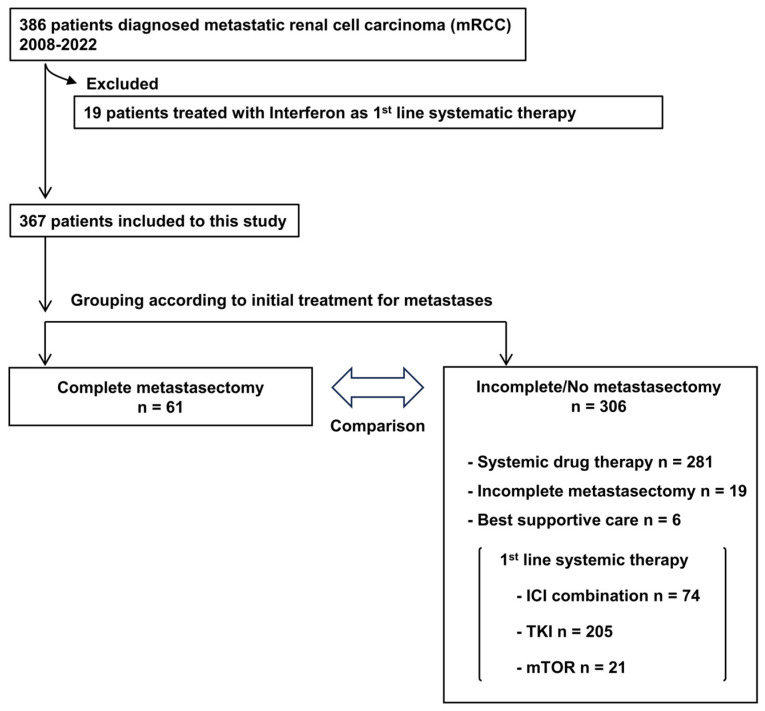
Flowchart of patient cohort dataset.

The patient selection process is delineated in Figure 1, wherein 19 patients who received cytokines as the first-line systematic therapy were excluded from the initial cohort of 386 patients. Subsequently, 367 patients were eligible for the analysis. Of these, 61 patients underwent CM as the initial intervention for metastatic lesions. Contrastingly, the remaining 306 patients received alternative treatments: 281 underwent systemic therapy, 19 received an incomplete metastasectomy, and 6 patients were subjected to best supportive care. The nineteen patients undergoing an incomplete metastasectomy subsequently received systemic treatment. Primary systemic treatment consisted of ICI combination therapy in 74 patients (ICI/ICI: 33 patients, ICI/TKI: 40 patients), TKI monotherapy in 205 patients and mammalian target of rapamycin inhibitor (mTORi) in 21 patients.

Table 1 summarizes the clinicopathological characteristics of the patients and compares the two groups before and after PSM: patients receiving CM (CM group) and those not receiving CM (non-CM group). Factors such as age, time from RCC diagnosis to treatment, surgical resection rate of the primary site, IMDC risk classification, metastatic site and number of metastases were significantly different between the two groups before PSM, while adjustment using PSM obtained a closely balanced distribution of the baseline covariates between the two groups.

### 3.2. Impact of Complete Metastasectomy on Oncological Outcomes

**Figure 2:**
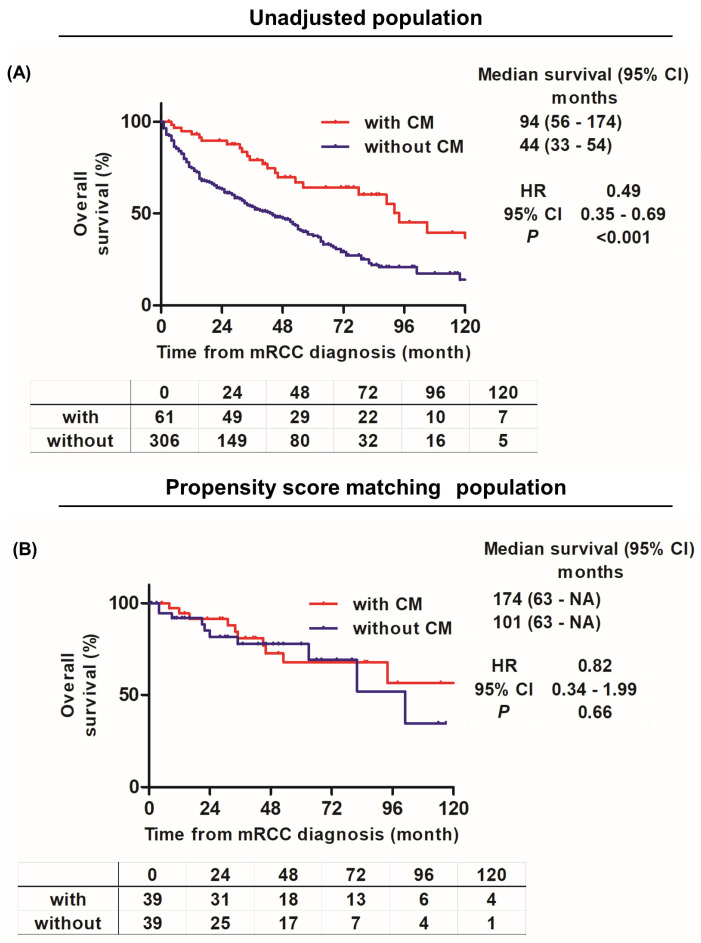
Kaplan–Meier curves before (**A**) and after (**B**) propensity matching of comparison of patients with or without complete metastasectomy in terms of overall survival.

The comparison of the OS curves of the unadjusted two groups revealed significantly longer OS in the CM group than in the non-CM group (*p* < 0.001 hazard ratio [HR] 0.49, 95% confidence interval [CI] 0.35–0.69) (Figure 2A). However, the comparison of the PSM-adjusted groups showed no significant difference in OS (Figure 2B).

### 3.3. Prognostic Factors in Patients with Complete Metastasectomy

**Figure 3:**
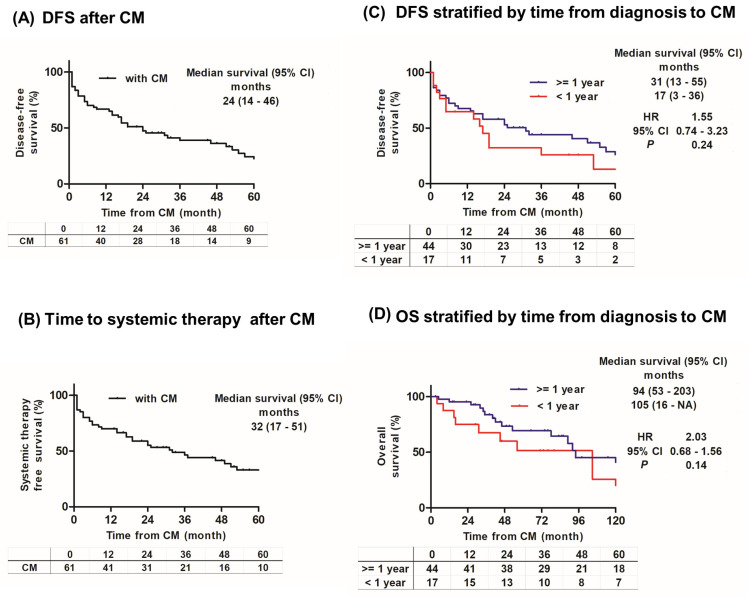
Kaplan−Meier curves after complete metastasectomy (CM). (**A**) Disease−free survival (DFS) curves post CM. (**B**) Time to systemic therapy from CM. (**C**) DFS after CM by time of metastasis identification (≥1 year or <1 year from renal cell carcinoma diagnosis). (**D**) Overall survival after CM by time of metastasis identification (≥1 year or <1 year from renal cell carcinoma diagnosis).

Of the 61 patients, 41 (67%) had disease recurrence after CM, with a median DFS of 24 months (95%CI, 14–46) (Figure 3A). The median systemic therapy-free survival from CM was 32 months (95%CI, 17–51) (Figure 3B). Next, the 61 patients were divided into two groups according to the time from their initial diagnosis to CM: 1 year and 1 year >. We compared the DFS and OS between patients receiving CM 1 year after their initial diagnosis and those receiving CM at 1 year > after their diagnosis. The time from the initial diagnosis to CM was not significantly associated with DFS and OS (Figure 3C,D).

Lastly, we explored the possible factors associated with DFS and OS after CM using a multivariate Cox hazard regression model (Table 2). Notably, the IMDC risk categories of intermediate and poor risk of the IMDC risk stratification and the presence of multiple metastatic sites were identified as independent poor prognostic factors for DFS. No independent prognostic factors were identified for OS in the multivariate analysis, while univariate analysis revealed that a higher age (>70 years-old) and the presence of metastasis in the lymph nodes, bones and brain were associated with a shorter OS.

## 4. Discussion

We observed that CM of mRCC was associated with improved OS compared with incomplete CM or no CM in the TKI/ICI era. This association decreased significantly after adjustment for age, primary site of pathology, IMDC risk classification, timing, location and number of metastases but showed no difference in OS compared to immediate systemic drug therapy. Lyon et al. [18] reported that in a cohort of 158 CM and 428 no or incomplete CM, the CM group had a significantly longer OS than the no or incomplete CM group, even after a background adjustment for age, gender, timing, and number and site of metastases, but not when adjusted for IMDC risk classification. A larger sample size may provide a clearer perspective. In addition, in recent years, combination therapy centered on ICIs has shown superiority and effectiveness in terms of long-term results compared to sunitinib alone, and the outcomes of mRCC are improving [19,20,21,22,23]. Our cohort included 24% new-age ICI combination therapies, which contributed to the improved prognosis of the non-CM group, suggesting that they may have led to the loss of superiority of the CM group. In any case, given that the median DFS after CM was 24 months, this indicates that even in the TKI/ICI era, long-term drug therapy may be avoided if the right patients are selected for CM treatment, and that CM has a potential beneficial role in the management of mRCC. Furthermore, in our cohort, there was no statistically superior difference in the oncological outcome between the potentially higher oncological grade group that relapsed concurrently or within one year and the group that relapsed after one year, but with median DFS rates of 17 and 31 months, differences could be seen if sample sizes were increased.

In reference to clinical trials evaluating postoperative adjuvant therapy for kidney cancer, including M1NED cases, such as IMmotion101 [17] and KEYNOTE564 [15], our study’s sample size may be smaller, yet it demonstrates comparable treatment outcomes. IMmotion101 also included CM for recurrence after one year after surgery, and the median time to recurrence was 30 months. Furthermore, in KEYNOTE564, the median DFS in the synchronous or recurrence within 1 year group without adjuvant therapy after CM (placebo) was 11.6 months. One of the findings of our current study is that patients with an IMDC risk classification of intermediate or poor risk and patients with multiple metastases are more likely to experience early recurrence after CM. These patients may be more likely to benefit from adjuvant pembrolizumab in M1NED cases. However, there is still debate as to whether adjuvant pembrolizumab should be administered after CM or whether ICI combination therapy should be administered from the beginning in these high-risk cases, and this will become more important in the future. Large-scale research may be required in the future.

Another advantage of CM is that the oncological potential of metastases can be estimated through pathology. Psutka et al. [24] reported that the pathology of metastatic foci does not necessarily match that of the primary tumor. They reported that the concordance rate of histological subtype and sarcomatoid differentiation was high, but the discordance rate of malignancy and coagulative necrosis between primary and metastatic tumors varied. Based on these, Pessoa et al. [25] used a part of the Leibovich score [26] for pathological findings in metastatic resection sites to examine possible predictors of recurrence and found that the metastatic site grading and necrosis were useful in predicting recurrence. These findings may help balance the benefit–risk balance of adjuvant pembrolizumab after M1NED surgery and inform decision making.

On the other hand, the safety considerations surrounding metastasectomy merit careful attention. Meyer et al. [27] conducted an extensive analysis comprising 1102 cases of metastasectomy, revealing an overall complication rate of 45.7%, with a noteworthy incidence of major complications (Clavien III-V) observed in 27.5% of cases. Notably, the resection of liver lesions emerged as a significant predictor of a heightened complication risk, with an odds ratio of 2.59 (95% confidence interval: 1.84–3.62, *p* < 0.001) compared to other metastatic sites. A major limitation of our study is that it was not possible to examine complications related to metastasectomy, as there were no reports of such complications like in Meyer’s study. Moreover, insights gleaned from Dudani et al.’s analysis [28] of the IMDC underscore the nuanced prognostic implications of the metastatic site in ccRCC. Their findings were that the prognosis of ccRCC varied according to the site of metastasis, with pleural metastases exhibiting a median overall survival (OS) of 16 months (95% CI: 13.7–18.8 months) compared with a median OS of 50 months (95% CI: 41.1–55.5 months) being observed in pancreas metastasis. Such granular prognostic stratification based on the metastatic site holds considerable relevance in informing the judicious selection of candidates for metastasectomy interventions. Furthermore, thermal ablation and radiotherapy are other options for the therapeutic armamentarium for mRCC. Noteworthy outcomes from a single-center series, comprising 84 patients harboring a total of 175 mRCC lesions, attest to the efficacy of stereotactic ablative radiotherapy, yielding an impressive local control rate of 91% at one year post-treatment. Encouragingly, the incidence of grade 3 or higher long-term toxicity was scant, observed in merely 3% of cases [29]. Moreover, a comprehensive synthesis of existing research on stereotactic radiotherapy corroborates the favorable local control rates that have been achieved across both intracranial and extracranial mRCC lesions, underscoring the therapeutic utility and safety profile of this modality [30].

The small sample size and the lack of assessment of safety aspects and metastatic pathology are major limitations, as has been discussed. Selection bias due to the background of the attending physician and patient in the choice of CM is another possible limitation. To address this shortcoming, a randomized trial would be ideal. However, given the relative rarity of this condition, it is unlikely that trials will be completed in the near future. Therefore, this study adjusted for background factors to further reduce the impact of selection bias, but real-world data are likely to be important, as CM should be selected for a very select group of cases in the first place. Clinicians should therefore rely on such observational data to inform their practice. In addition, the method and duration of follow-up after CM, treatment at relapse and treatment of the non-CM group were left to the judgment of each attending physician and were not consistent.

## 5. Conclusions

This study underscores the potential of CM in mRCC management during the TKI/ICI era. However, limitations including safety evaluation gaps and sample size constraints necessitate cautious interpretation. Real-world data remain pivotal for informed clinical decision making due to the difficulties in conducting randomized trials.

## Figures and Tables

**Table 1:** Clinicopathologic variables of patients with metastatic renal cell carcinoma and comparison between patients with and without complete metastasectomy.

Variables		Unadjusted Population		*p* Value	PSM Population		*p* Value	SMD
		Complete Metastasectomy			Complete Metastasectomy			
		No n = 306	Yes n = 61		No n = 39	Yes n = 39		
Age (years old), Median (IQR)		69 (63–77)	65 (59–72)	0.01 ‡	66 (59–74)	64 (57–72)	0.43 ‡	0.18
	70≤	168 (54.9%)	25 (41.0%)	0.05 †	14 (35.9%)	14 (35.9%)	1.00 †	<0.001
Sex	Male	228 (74.5%)	42 (68.9%)	0.42 †	30 (76.9%)	28 (71.8%)	0.80 †	0.12
	Female	78 (25.5%)	19 (31.1%)		9 (23.1%)	11 (28.2%)		
Time from diagnosis to treatment	Yes	100 (32.7%)	44 (72.1%)	<0.01 †	29 (74.4%)	27 (69.2%)	0.80 †	0.11
Asynchronous, ≥1 year	No	206 (67.3%)	17 (27.9%)		10 (25.6%)	12 (30.8%)		
Surgical removal of primary organ	Yes	238 (77.8%)	61 (100%)	<0.01 †	39 (100%)	39 (100%)	NA	<0.001
	No	68 (22.2%)	0 (0%)		0 (0%)	0 (0%)		
IMDC risk classfication	favorable	52 (17.0%)	23 (37.7%)	<0.01 †	16 (41.0%)	15 (38.5%)	0.51 †	0.32
	intermediate	179 (58.5%)	32 (52.5%)		22 (56.4%)	20 (51.3%)		
	poor	75 (24.5%)	6 (9.8%)		1 (2.6%)	4 (10.3%)		
Histology	ccRCC	197 (64.4%)	48 (88.7%)	0.23 †	37 (94.9%)	33 (84.6%)	0.26 †	0.34
	Non-ccRCC	47 (15.4%)	13 (21.3%)		2 (5.1%)	6 (15.4%)		
	Sarcomatoid change (+)	27 (8.9%)	7 (11.5%)	0.63 †	4 (10.3%)	3 (7.7%)	0.63 †	0.09
	NA	62 (20.3%)	0 (0%)					
Fuhrman Grade	G1,G2	106 (34.6%)	29 (47.5%)	0.28 †	20 (51.3%)	22 (56.4%)	1.00 †	0.10
	G3,G4	127 (39.1%)	23 (37.7%)		19 (48.7%)	17 (43.6%)		
	NA	73 (23.9%)	9 (14.8%)					
Metastatic sites or target lesions	Lymph node	106 (34.6%)	8 (13.1%)		3 (7.7%)	6 (15.4%)	0.48 †	0.24
	Lung	172 (56.2%)	34 (55.7%)		26 (66.7%)	22 (56.4%)	0.49 †	0.21
	Liver	25 (8.2%)	2 (3.4%)		25 (8.2%)	2 (3.4%)	1.00 †	<0.001
	Bone	69 (22.5%)	13 (21.3%)		6 (15.4%)	7 (17.9%)	1.00 †	0.07
	Brain	5 (1.5%)	6 (9.8%)		1 (2.6%)	3 (7.7%)	0.62 †	0.23
	Others	87 (28.4%)	17 (27.4%)		5 (12.8%)	6 (15.4%)	1.00 †	0.07
Number of metastases		230 (75.2%)	51 (83.6%)	0.19 †	35 (89.7%)	34 (87.2%)	1.00 †	0.08
	2≤	76 (24.8%)	10 (16.4%)		4 (10.3%)	5 (12.8%)		
Follow-up period (month), median (IQR)		32 (9–49)	58 (28–80)		41 (14–60)	54 (28–76)		

PSM, propensity score match; IQR, interquartile range; SD, standard deviation; SMD, standardized mean difference; ccRCC; clear cell renal cell carcinoma; eGFR, estimated glomerular filtration rate; NA, not available; G, grade; ‡, Mann–Whitney test; †, Fisher’s exact test.

**Table 2:** Multivariate Cox regression analyses of clinical and pathological features for progression-free survival and overall survival after complete metastasectomy (* statistically significant).

Variables		Progression-Free Survival			Overall Survival		
		Multivariate Analysis			Multivariate Analysis		
		HR	95% CI	*p* Value	HR	95% CI	*p* Value
Age	≥ 70 vs. <70				2.42	0.90–6.51	0.08
Time from diagnosis to treatment	<1 year vs. ≥1 year				0.35	0.09–1.32	0.12
IMDC	Intermediate/Poor vs. Favorable	4.37	1.05–4.37	0.036 *	2.31	0.57–9.33	0.24
Fuhrman grade	3, 4 vs. 1, 2				2.09	0.63–6.96	0.23
Site of metastasectomy							
Lymph node	Yes vs. No				2.31	0.54–9.96	0.26
Lung	Yes vs. No				1.33	0.33–5.32	0.68
Liver	Yes vs. No						
Bone	Yes vs. No	0.86	0.39–1.89	0.71	3.21	0.75–13.7	0.12
Brain	Yes vs. No				2.1	0.63–6.96	0.38
Number of metastasis	Multi vs. Mono	3.74	1.61–8.71	0.002 *			

HR = hazard ratio; CI = confidence interval; IMDC = international metastatic RCC database consortium.

## Data Availability

The data presented in this study are available on request from the corresponding author.
